# McKillip

**Published:** 1896-04

**Authors:** 


### McKILLIP VETERINARY COLLEGE.

Having no senior class for the present year, the closing exercises on the 25th of March consisted of addresses by members of the faculty to the students on subjects pertaining to studentship and the profession.

